# Portal vein thrombosis after laparoscopic appendectomy for acute appendicitis: A case report

**DOI:** 10.1097/MD.0000000000042068

**Published:** 2025-04-04

**Authors:** İsmail Tirnova, Maya Gasimova, Behlül Igus, Alpay Yeşilaltay, Derya Kaşkari, Saime Ramadan, Ahmet Serdar Karaca

**Affiliations:** a Department of General Surgery, Başkent University School of Medicine, İstanbul, Turkey; b Department of Radiology, Başkent University School of Medicine, İstanbul, Turkey; c Department of Internal Medicine, Division of Haematology, Başkent University School of Medicine, İstanbul, Turkey; d Department of Internal Medicine, Division of Rheumatology, Başkent University School of Medicine, İstanbul, Turkey; e Department of Pathology, Başkent University School of Medicine, İstanbul, Turkey.

**Keywords:** acute appendicitis, case report, laparoscopy, portal vein, thrombosis

## Abstract

**Rationale:**

Portal vein thrombosis (PVT) has a complex pathophysiologic pathway and may cause life-threatening clinical complications. Malignancies, hepatic cirrhosis, auto-immune disorders, previous splenectomy, and other causes of thrombocytosis (over 1,000,000/mL) are the most common causes of PVT. On the other hand, hematologic disorders and infectious processes in the abdominal cavity may cause PVT uncommonly. We present a case of PVT following acute appendicitis and laparoscopic appendectomy in this report.

**Patient concerns:**

A 32-year-old male was admitted to our emergency room due to lower quadrant pain and vomiting. Acute appendicitis was diagnosed and after a routine laparoscopic appendectomy, the patient was discharged. The patient was admitted to the emergency room with nonspecific epigastric pain on postoperative day 30.

**Diagnoses:**

Portal vein thrombosis was diagnosed by computed tomography. Hematologic investigations revealed a homozygous mutation of the methylene tetrahydrofolate 1298 gene.

**Interventions:**

Immediate low-molecular-weight heparin administration was initiated. The gastrointestinal system council and interventional radiology team opted for a medical approach and converted the low-molecular-weight heparin to apixaban.

**Outcomes:**

The computed tomography revealed the complete resolution of the thrombus on postoperative day 100.

**Lessons:**

Laparoscopic appendectomy can be complicated by portomesenteric axis thrombosis. When unusual findings are encountered during the postoperative follow-up period, rapid and detailed examinations should be performed.

## 1. Introduction

Portal vein thrombosis (PVT) has a complex pathophysiologic pathway and may cause life-threatening clinical complications, especially in delayed-diagnosed patients.^[1]^ Malignancies, hepatic cirrhosis, auto-immune disorders, previous splenectomy, and other causes of thrombocytosis (over 1,000,000/mL) are the most common causes of PVT.^[2]^ On the other hand, hematologic disorders and infectious processes in the abdominal cavity may cause PVT uncommonly. Rarely, there have been several publications regarding portomesenteric thrombosis as a complication of acute appendicitis in adults, and the pediatric age group.^[3–5]^ Early recognition is an indispensable factor for successfully managing PVT following acute appendicitis. Otherwise, acute and chronic complications of PVT may cause devastating outcomes in both medical and financial manners.

We present a case of PVT following acute appendicitis and laparoscopic appendectomy in this report.

## 2. Case presentation

**Figure 1.**
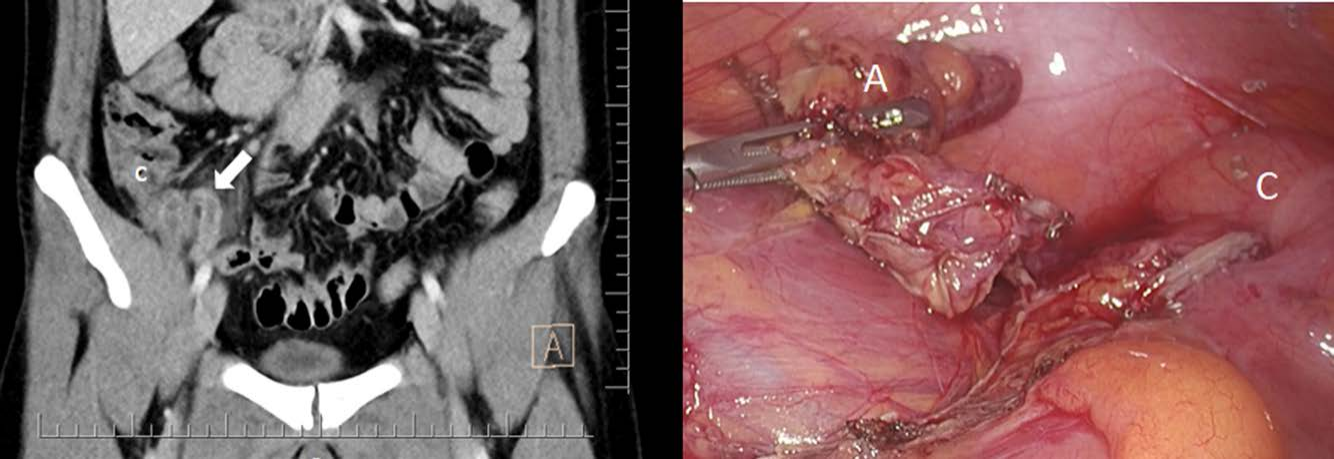
Computed tomography scans performed at the external center during the first emergency service admission of the patient, and intraoperative scene. The blind-ended and thickened-walled intestinal structure (white arrow). C = caecum, A = appendix.

**Figure 2.**
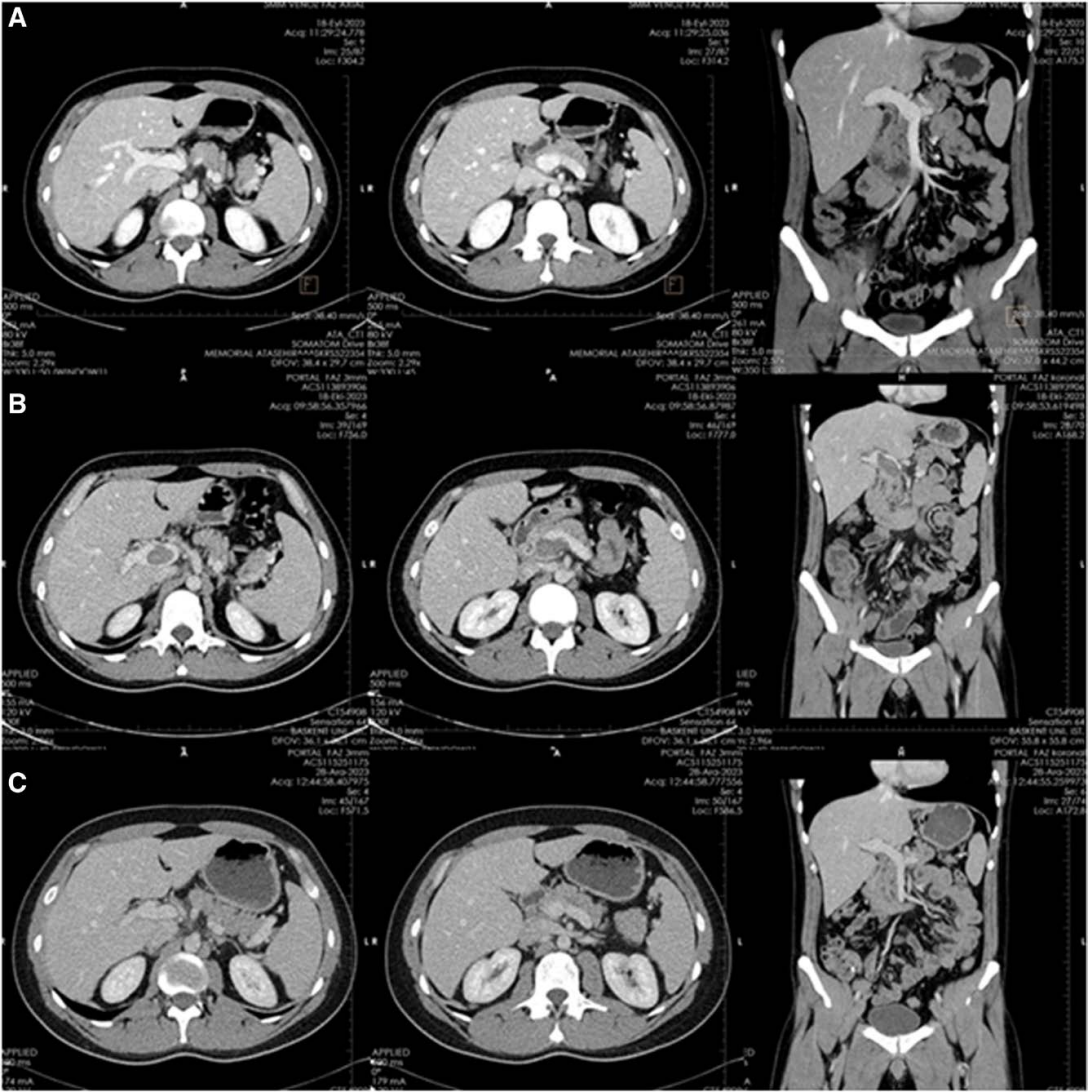
(A) The retrospective analysis of computed tomography (CT) scans performed at the external center during the first emergency service admission of the patient. (B) The CT scans of POD 30. (C) The CT scans of the POD 100.

A 32-year-old engineer was admitted to our emergency room (ER) due to lower quadrant pain and vomiting. He had varicocelectomy in his history. He had no ongoing medication. His pain had started about 40 hours before his admission to our ER. The pain was initially located in the epigastric part of his abdomen in the early hours and then migrated to the right lower quadrant. The patient reported anorexia, nausea, and vomiting. The vital signs were completely normal; blood pressure of 132/83 mm Hg, heart rate of 79 beats per minute, respiratory rate of 17 breaths per minute, temperature of 37.2 °C, and peripheral oxygen saturation of 99% on room air. Physical examination revealed tenderness, guarding, and rebound for the right lower quadrant. Other quadrants were normal. Laboratory evaluations revealed normal results except a mildly elevated white blood cell count of 12.4 (10^3^/µL). C-reactive protein was 78 mg/L. The platelet count was 158,000/µL. The international normalized ratio was 1.12. Liver and renal functions were normal. The patient was evaluated at another hospital before our center and a contrast-enhanced computed tomography (CT) scan was performed there. CT scans were evaluated with the radiology team at our hospital and revealed a blind-ended and thickened-walled intestinal structure beside the cecum (Fig. 1). The other parts of the scans were completely normal (Fig. 2A).

The patient was started on intravenous fluid hydration. Pain control was established with intravenous morphine administration. Prophylactic antibiotic coverage was established with 2 gr cefazolin and 500 mg metronidazole. Laparoscopic appendectomy was performed via 3 trocars uneventfully (Fig. 1). There was no need to use a surgical drain. The appendectomy specimen was sent to the pathology department. Acute purulent appendicitis was diagnosed. The postoperative period was also uneventful. Oral intake was started at the sixth postoperative hour and was well tolerated. The patient was discharged on postoperative day (POD) 1 after normal laboratory results and a normal physical examination of 3 trocar sites. The patient was started on oral ciprofloxacin, metronidazole, and paracetamol by discharge.

The first postoperative outpatient control was uneventfully on POD 3. There was no surgical site infection or wound dehiscence. Oral intake was normal. The second postoperative outpatient control was also uneventfully on POD 10 and the patient’s follow-up was abandoned.

The patient was readmitted to our ER on POD 30 with nonspecific epigastric pain. The vital signs were normal; blood pressure of 127/78 mm Hg, heart rate of 69 beats per minute, respiratory rate of 18 breaths per minute, temperature of 36.8 °C, and peripheral oxygen saturation of 99% on room air. Physical examination was entirely normal. Laboratory evaluations revealed normal results except for a mildly elevated liver function test. C-reactive protein was 22.5 mg/L. The platelet count was 158,000/µL. INR was 1.12. Renal functions were normal. Contrast-enhanced CT (Fig. 2B) revealed a Yerdel Grade III PVT.^[6]^ The patient was immediately hospitalized. Low-molecular-weight heparin (LMWH) was started. The preoperative CT scans were analyzed retrospectively. There was no thrombosis in the portal vein at first administration for acute appendicitis (Fig. 2A). The patient was discussed in the Gastrointestinal System Council. The interventional radiology team, which experienced over 800 liver transplant cases, opted for a medical approach and did not offer an endovascular approach for the thrombus. LMWH was converted to apixaban by the council. On the other hand, rheumatologic and hematologic investigations were performed (Table 1). After 4 days of hospitalization, the patient was discharged uneventfully with apixaban coverage.

**Table 1:** Laboratory findings when portal vein thrombosis was diagnosed.

Parameter	Result	Normal range	Unit
Hemoglobin	13.1	13.5–18	g/dL
Platelet	213	150–450	10^3^/µL
White blood cell (WBC)	6430	4–11	10^3^/µL
Creatinin	0.72	0.6–1.2	mg/dL
ALT	71	0–55	U/L
AST	43	5–34	U/L
GGT	63	0–55	U/L
ALP	73	40–150	U/L
Total bilirubin	0.66	0.2–1.2	mg/dL
C reactive protein (CRP)	22.5	0–5	mg/L
INR	1.21	0.8–1.2	–
Antithrombin III	19	22–39	mg/dL
Protein C activity	95	61–162	%
Factor V	77	50–150	%
Kompleman C3	1.25	0.82–1.85	g/L
Kompleman C4	0.343	0.15–0.53	g/L
Sedimentation	12	0–20	mm/hr
ANA (antinuclear antikor)	Negative	Negative	
Lupus anticoagulant (with confirmation)	1.21	Negative: <1.20	
Anticardiolipin Ig M	<2	0–12	GPL/mL
Anticardiolipin Ig G	<2	0–12	GPL/mL
HLAB51	Negative		
Anti beta-2 glycoprotein IgG	2.57	–	U/mL
Anti beta-2 glycoprotein IgM	2.31	–	U/mL
Rheumatoid Factor	0.01	0–30	IU/mL
MTHFR c1298A>C	Homozygous mutant		
MTHFR c677C>T	Normal		

ALP = alkaline phosphatase, ALT = alanine aminotransferase, AST = aspartate aminotransferase, GGT = gamma-glutamyl transpeptidase, HLA = human leukocyte antigen, Ig = immunoglobulin, INR = international normalized ratio, MTFR = methylenetetrahydrofolate reductase.

The patient was completely asymptomatic after apixaban started. The contrast-enhanced CT revealed the complete resolution of the PVT on POD100 (Fig. 2C). The patient is still under apixaban prophylaxis and is still asymptomatic (POD150).

## 3. Discussion

Malignancies, rheumatologic, and hematologic disorders are well-known reasons for portal vein thrombosis among adults. Usually, the patients have disease-specific symptoms and biochemical or radiologic findings before the PVT occurs in these cases, so PVT is diagnosed during routine follow-up in most of these patients. Moreover, intraabdominal infections may be the main reason for PVT in healthy individuals. Diverticulitis and acute appendicitis may be the trigger mechanism for this entity. Some authors have provided that the thrombosis of the portomesenteric axis occurred following an ascending thrombosis or the liberation of any septic thrombus to the portal venous structures from the initial infectious area.^[3]^ Diverticulitis is a chronic onset pathology and usually, patients have symptoms such as recurrent abdominal pain, distention, and constipation in their history. Acute appendicitis is another infective trigger for portomesenteric thrombosis. Only limited reports concerning PVT after acute appendicitis are available in the literature.^[7–11]^ Schmutz et al reported 6 adult cases as their experience and Yoon et al reported nineteen children and adolescents in their current review.^[3,4]^ A review by James et al evaluated the portomesenteric thrombosis after laparoscopic surgical interventions.^[12]^ Eighteen patients were in that review, and only one was an appendectomy case. The other trigger surgical interventions for PVT were cholecystectomy, colectomy, Nissen fundoplication, and gastric bypasses. In our case, the interval between the diagnosis of appendicitis and the onset of abdominal pain leading to the diagnosis of portal vein thrombosis was 30 days. In other cases reported in the literature, although acute appendicitis and portal vein thrombosis may be diagnosed simultaneously, symptoms often appear within the first 2 weeks.^[7–11]^

Rheumatologic disorders were excluded in our case. According to rheumatologic questions, the patient didn’t fulfill the International Criteria for Behçet’s disease, which were developed in 2006, and the American College of Rheumatology and European Alliance of Associations for Rheumatology released updated classification criteria in 2023 for antiphospholipid syndrome.

Haematologic disorders are another reason for PVT. The gene referred to as methylene tetrahydrofolate reductase (MTHFR) is located on chromosome 1p36.3, and a mutation in this gene occurs when cytosine (C) is replaced by thymidine (T) at position 677 and when adenosine (A) is replaced by cytosine (C) at position 1298. MTHFR polymorphisms are divided into homozygous normal genotypes, heterozygous genotypes, and homozygous mutation genotypes.^[13]^ Particularly, the 677C>T mutant genotype is associated with high plasma homocysteine levels.^[14]^ Hyperhomocysteinemia resulting from mutations is considered an independent risk determinant for atherosclerotic vascular disease and venous thromboembolism.^[15]^ Elevated homocysteine levels are primarily atherogenic and prothrombotic, leading to intimal thickening, disruption of the elastic lamina, smooth muscle hypertrophy, significant platelet accumulation, and occlusive thrombi-containing platelets. The 1298A>C mutant genotype has also been reported to be associated with high plasma homocysteine levels.^[16]^ However, it has been reported that the 1298A>C mutant genotype can be clinically associated with thrombosis only if it occurs simultaneously with the 677C>T mutation.^[17]^ In our case, MTHFR 677 was normal, while MTHFR 1298 was a homozygous mutant. The coincidence of acute appendicitis and underlying homozygous mutant MTHFR gene may be responsible factors for the development of PVT in our case.

Management of an acute PVT may include anticoagulation, regional or systemic thrombolysis, thrombectomy, or a combination of these treatment options. The main goals are to prevent the thrombus’s extension, achieve recanalization of the portal vein, and prevent complications of chronic PVT.^[18]^ It is important to start anticoagulant treatment immediately as a noninvasive method in the first step. In the literature, only a moderate (38%) treatment response has been reported with oral anticoagulant treatments.^[19,20]^ Treatment duration varies depending on etiology and treatment response. In cases that are mildly symptomatic and diagnosed early, as in our case, early initiation of medical treatment may increase the chance of successful results. In our case, a complete response was achieved and no invasive techniques were required. However, due to the existing MTHFR mutation, continuing the treatment and switching to long-term prophylaxis is important to prevent recurrence and the development of other thrombotic events.^[21]^ In some cases, mutation may be diagnosed after a complication, as in our case. Once portal vein (PV) thrombus occurs in MTHFR mutant patients, it is recommended to switch to aspirin use at a dose of 100 mg/day for life, following direct oral anticoagulants use for 6 months.^[22]^

Medical treatment options include LMWH group drugs such as enoxaparin and dalteparin, classical oral anticoagulants such as warfarin, and direct oral anticoagulants such as rivaroxaban and apixaban.^[23]^ There is no significant difference between the groups in terms of treatment success, and patient-specific preferences can be made.

Endovascular treatment of acute PVT may be considered in patients with findings of imminent bowel infarctions or patients with contraindications to systemic anticoagulation. Several endovascular techniques can be performed, including catheter thrombectomy with PV catheterization and initial thrombolytic infusion. In patients with insufficient portal vein outflow after the catheter thrombectomy, thrombolytic therapy infusion can be applied for 24 to 48 hours with PV catheterization to resolve a thrombus.^[24]^ Also, endovascular thrombectomy with PV catheterization can be performed in case of persistent thrombus with prolonged anticoagulant treatment for up to 12 weeks. If remnant thrombus remains in the portal vein after thrombectomy and thrombolytic therapy, placement of a vascular stent may be beneficial in ensuring adequate portal flow.

Especially in complicated cases, transhepatic or transjugular PV catheterization and, if necessary, stent placement may be preferred.^[18,22]^ Rarely surgical and endovascular techniques are required to be combined in complicated cases and have been reported successful outcomes.^[20]^

## 4. Conclusion

In conclusion, laparoscopic appendectomy, one of the most frequently performed laparoscopic surgical interventions, can be complicated by portomesenteric axis thrombosis. When unusual findings are encountered during the postoperative follow-up period, successful results can be achieved in such dangerous complications with rapid and detailed examinations.

## Acknowledgments

The authors would like to thank Professor Fatih BOYVAT MD, for his valuable efforts during the management of the case.

## Author contributions

**Investigation:** Maya Gasimova, Saime Ramadan.

**Methodology:** Behlul Igus.

**Supervision:** Ahmet Serdar Karaca.

**Writing – original draft:** İsmail Tirnova, Alpay Yeşilaltay, Derya Kaşkari.

**Writing – review & editing:** Ahmet Serdar Karaca.
